# Supplementary material for: Conditional chemoconnectomics (cCCTomics) as a strategy for efficient and conditional targeting of chemical transmission
Source: eLife. 2024 Apr 30;12:RP91927. doi: 10.7554/eLife.91927 (PMC11060718; doi:10.7554/eLife.91927)
Supplement: Figure 3—source data 3. [file elife-91927-fig3-data3.zip › Figure 3 source data3 part 2.zip!/Figure 3-Source data 3-part2/1062-13-CRISPResso_Report_jt28fhr9.zip!/CRISPResso_on_1062_16/3b.gcaggaaatgagctacctac.Insertion_deletion_substitutions_size_hist.pdf]

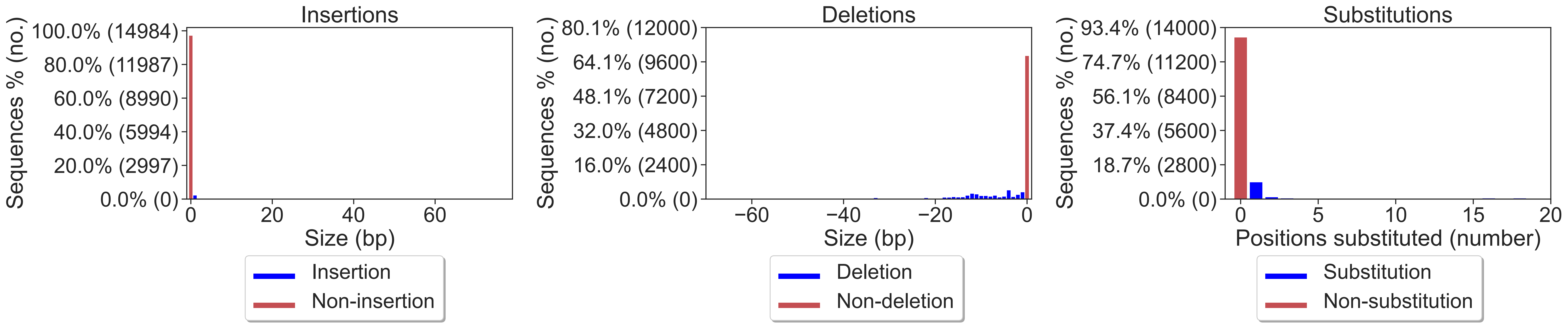

Insertions
Sequences % (no.)
100.0% (14984)
80.0% (11987)
60.0% (8990)
40.0% (5994)
20.0% (2997)
0.0% (0)
0
20
40
60
Size (bp)
Insertion
Non-insertion
Deletions
Sequences % (no.)
80.1% (12000)
64.1% (9600)
48.1% (7200)
32.0% (4800)
16.0% (2400)
0.0% (0)
−60
−40
−20
0
Size (bp)
Deletion
Non-deletion
Substitutions
Sequences % (no.)
93.4% (14000)
74.7% (11200)
56.1% (8400)
37.4% (5600)
18.7% (2800)
0.0% (0)
0
5
10
15
20
Positions substituted (number)
Substitution
Non-substitution
